# An experimental and numerical study of the flow and mass transfer in a model of the wearable artificial kidney dialyzer

**DOI:** 10.1186/1475-925X-9-21

**Published:** 2010-05-24

**Authors:** Edmond Rambod, Masoud Beizai, Moshe Rosenfeld

**Affiliations:** 1 BioQuantetics, Inc., 11731 Folkstone Lane, Los Angeles, CA 90077 USA; 2 School of Mechanical Engineering, Faculty of Engineering, Tel-Aviv University, Ramat Aviv, Tel-Aviv 69978, Israel

## Abstract

**Background:**

Published studies of the past decades have established that mass transfer across the dialyzer membrane is governed by diffusion, convection and osmosis. While the former is independent of the pressure in the liquids, the latter two are pressure dependent and are enhanced when the pressure difference across the membrane is increased. The goal of the present study is to examine the impact of pulsatile flow on the transport phenomena across the membrane of a high-flux dialyzer in a wearable artificial kidney (WAK) with a novel single small battery-operated pulsatile pump that drives both the blood and dialysate in a counter-phased manner, maximizing the trans-membrane pressure.

**Methods:**

Both in-vitro experimental and numerical tools are employed to compare the performance of the pulsatile WAK dialyzer with a traditional design of a single-channel roller blood pump together with a centrifugal pump that drives the dialysate flow. The numerical methods utilize the axisymmetric Navier-Stokes and mass transfer equations to model the flow in the fibers of the dialyzer.

**Results:**

While diffusion is still the dominating transport regime, the WAK pump enhances substantially the trans-membrane pressure and thus increases mass convection that might be as high as 30% of the overall transfer. This increase is obtained due to the design of the pulsatile WAK pump that increases ultrafiltration by increasing the trans-membrane pressure.

**Conclusions:**

The experimental and numerical results revealed that when pumping at similar flow rates, a small battery-operated pulsatile pump provides clearances of urea and creatinine similar as or better than a large heavy AC-powered roller pump.

## Background

The mechanisms of solute transport across dialyzer's membranes have been studied for more than half a century. Historically, it appears that pioneers of dialytic therapy were well aware of diffusive phenomena when they designed dialyzers based on counter-current exchangers [1,2]. Later on, convection was realized to be a primary mechanism in ultrafiltration and 'solvent drag' phenomena [3-5]. The factors influencing solute transport across semi-permeable membranes have been summarized by Ronco *et al *[6]. Blood flow rates greatly affect the clearance of small solutes such as urea, but larger solutes are affected mainly by ultrafiltration rates.

The shortcomings of steady flow dialyzer, especially in promoting protein adsorption, are well known and therefore time-dependent flow solutions have been suggested. Most notably, the push/pull hemodiafiltration has been studied quite extensively. The effect of pulsatile blood flow on the filtration rate of hollow fibers has been studied by various researchers [4,7-9] while adsorption of proteins has been also considered, e.g. [10,11]. These studies indicate clinically interesting and effective blood purification outcomes enhanced by convective solute removal and protein washout by the push-pull mechanism. A push-pull hemodiafiltration (HDF) device provides rapidly alternating forward-backward filtration [8,9]. This mechanism leads to alternate flow of body fluid and sterile pyrogen-free dialysate across a high flux hollow-fiber membrane, enhancing overall performance of the dialyzer. The main drawbacks of these push-pull HDF devices are the necessity of a disposable blood reservoir bag to prevent flow variation, and the difficulty in maintaining the trans-membrane pressure (*TMP*) that may lead to collapse of hollow fibers during backfiltration. The remedy to these problems has been suggested to be the use of volume controllers for ultrafiltrate removal and rigid synthetic hollow fibers such as polyacrylonitrile, polysulfone, and polyamide [8,9].

The flow and mass transfer processes in dialyzers have been studied computationally by employing either 1-D lumped models [e.g. [12]] or by solving the Navier-Stokes equations in 2-D or 3-D models e.g. [13-16], including models of hollow fibers and the surrounding shell. However, in the multi-dimensional cases, only conventional steady flow dialyzers have been modeled. Computational studies relevant to pulsating flow dialyzers have not been published.

The wearable artificial kidney (WAK) developed in the past several years [17-21] is a light-weight belt-type battery-operated device which utilizes the advantages of pulsatile low flow rate of blood and dialysate (40-80 ml/min) to provide around-the-clock dialysis treatment to patients with end-stage renal disease. The present study is aimed at exploring the impact of pulsatile flow of low flow rate on the transport of small molecule solutes in a high-flux dialyzer. Comparative experimental studies and numerical simulations have been deployed to clarify and verify the role of parameters influencing mass transport phenomena across the membrane of the WAK dialyzer with counter-phased pulsatile flow. The results are compared with a conventional dialyzer. Since the WAK is working around-the-clock, a significantly lower flow rate can be employed. None of the previous experimental or computational studies have considered this regime of parameters and operational modes.

## Methods

### Experimental Apparatus and Methods

The main pulsatile pump of the WAK uses a 3-Watt DC micro-motor (Faulhaber, Schoenaich, Germany). The mechanical assembly of the pump has been redesigned to accommodate an oscillating mechanism, which in conjunction with a custom-made dual-ventricle flow cartridge allows counter-phased, simultaneous pulsatile flows of both blood and dialysate at controllable rates of 40-80 ml/min, [17-21]. When one channel is propelling fluid out of its compliant chamber (very much as in "systole"), the other one is filling the compliant ventricle ("diastole"), creating a peak pressure in one channel at the same time the pressure in the other channel is at its lowest level.

To compare the effect of pulsatile flow generated by the dual-ventricle pump, a roller pump (Minipump™, MINNTECH renal systems, Minneapolis, MN), which is similar to conventional hemodialysis machine blood pumps, is used to drive the blood flow, while a centrifugal pump is employed to drive the dialysate flow. In both cases, Multiflow 60 AN69 HF hollow fiber dialyzer (Hospal Industrie, Meyzieu, France) is used.

The WAK pump measures 10 × 7 × 5 cm, weighs 380 gm and produces 95 ml/min (peak 350 ml/min) each of blood and dialysate at 110 cycles/min and a stroke volume of 0.8 cm^3^. The Minipump measures 30 × 15 × 15 cm, weighs 4650 gm and produces 95 ml/min (peak 120 ml/min) of blood at 13 cycles/min and a stroke volume of 8 cm^3^. The centrifugal pump (Powerhead, Meiko, Taichung, Taiwan) measures 8 × 7 × 7 cm and weighs 65 gm. Additional details on the experiment setup used can be found in Ref. [20].

**Figure 1:**
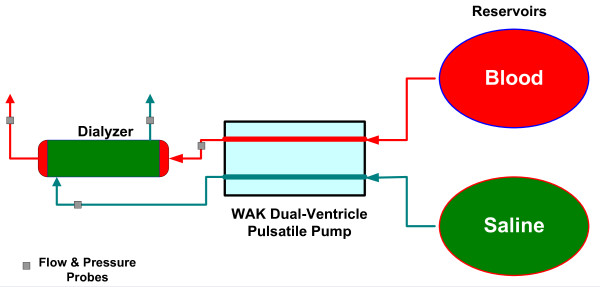
**A schematic description of the WAK experimental setup**.

Two series of experiments are performed, one using the WAK pulsatile pump, the other using the roller and centrifugal pumps combination. Figure 1 shows the conceptual schematics of the test setup. While both the WAK and the roller pumps push blood *into *the dialyzer, the dual-ventricle pump in the WAK pulls dialysate *out *of the dialyzer whereas a centrifugal pump is used to pump the dialysate through the dialyzer in the second setup. Either porcine blood or imitation blood (isotonic saline) are used. In the cases with the porcine blood, approximately 5 liters of heparinized porcine blood are prepared with a hematocrit of ~30%, following Protocol P00504 (Porcine Blood Handling), and it is boosted with BUN and creatinine of about 60 and 10 mg/dL, respectively. The blood is mixed gently every five minutes to keep it homogenous. In the beginning of the first experiment, the blood is re-circulated for one minute through the primary reservoir to ensure that all components of the system work properly without loss of blood. Following this brief period of one minute, the blood line is altered in a way that mimics the return of blood to the patient to maintain a reasonable trans-membrane pressure (*TMP*). The initial concentrations of urea and creatinine in the reservoir are measured and recorded at this point. Standard isotonic saline is used as the dialysate.

Both the blood and dialysate returns are located at levels of the respective reservoir containers. Blood return is collected for use in roller pump experiments whereas dialysate return is discarded. In some tests, dialysate return passed through powder-filled canisters to generate an afterload that increases the pressure level at the blood outlet. Ultrafiltration is drawn in some of the experiments. In addition, a pH meter electrode is placed in the dialysate circuit at the exit site from the sorbent system and set to trigger a sodium bicarbonate infusion to the dialysate prior to its entrance into the dialyzer to maintain the pH of the dialysate in the dialyzer at approximately 7.4.

Flow rates and pressure are measured simultaneously at the inlets and outlets of the dialyzer's compartments. Four ultrasonic flow meters and probes (Transonic Systems, Ithaca, NY), and four pressure sensors (Merit Medical, South Jordan, UT) are employed, see Figure. 1. A LabView virtual instrument (National Instruments, Austin, TX) is used to record the flow and pressure. i-STAT Portable Clinical Analyzer (Abbott, East Windsor, NJ) and corresponding cartridges are used for in-situ concentration measurement of solutes in both the blood and dialysate flows. The i-STAT readings are subsequently corroborated by an outside lab as the readings for dialysate are only qualitative. Each experiment of WAK pump settings of 6.0, 7.5, 9.0 and 12 Volts is carried out for a 15 minutes period. The mean flow rate for these settings is approximately 40, 50, 60 and 80 ml/s, respectively. For each experiment, samples are drawn from the blood and dialysate exiting the dialyzer in one and a half minute intervals. The concentrations of urea and creatinine in these samples, 10 per experiment, are analyzed by an outside lab. The flow conditions for the roller pump are adjusted to satisfy an approximately equal inflow blood as with the WAK pump. Similarly, the flow conditions with the centrifugal pump are adjusted to satisfy an approximately equal inflow saline as with the WAK pump.

### The Numerical Model

**Figure 2:**
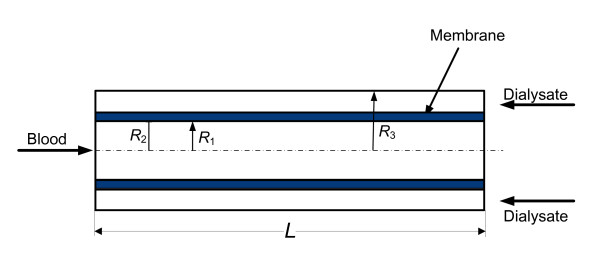
**The computational domain**.

The Multiflow M60 hemofilter consists of approximately 4400 AN69 hollow fibers with an inner radius of 120 μm, a thickness of 50 μm and a length of 15 cm. The inner diameter of the hemofilter is 33 mm. As a first approximation, the hollow fibers are assumed to be distributed uniformly inside the hemofilter. The array of hollow fibers is represented by a single hollow fiber, where the blood flows in one direction, and the surrounding shell, where the dialysate flows in the counter direction. A further simplification assumes that this basic unit is axisymmetric with an outer radius of 225 μm. Figure 2 illustrates the domain used in the model. The flow in the shell is found to be non-uniform [15]. Yet, since the aim of the present investigation is to quantify mass transfer processes, the present simplifications of the geometry are reasonable. The domain of computation is composed of three sub-domains (Figure 2), similar to previous numerical studies [14-16]. Two domains are free-flow domains: the blood flow in the hollow fiber and the dialysate flow in the surrounding shell. The third sub-domain is the porous membrane domain.

The continuity equation is(1)

where *ρ *is the mixture density and  is the velocity (in the membrane region, the superficial velocity is used). The momentum conservation (Navier-Stokes) equation for a laminar Newtonian flow is:(2)

where *P *is the pressure, μ is the molecular viscosity and  is a source term that vanishes everywhere except in the porous membrane domain.

The membrane is modeled as a porous rigid medium using the *Brinkman *approximation, [22]. The radial momentum source term is composed of two parts: a viscous (Darcy) loss term and a term rising from the osmotic pressure:(3)

where *v *is the radial velocity component, *k *is the permeability and *π *is the osmotic pressure. The osmotic pressure is mainly produced by proteins. For small molecule solutes, as considered in the present study, the osmotic pressure is negligible. Neglecting the inertial and viscous terms in the membrane region, the radial momentum equation can be integrated to yield(4)

The difference (Δ) is taken between the two membrane surfaces (with the same axial coordinate), *h *is the thickness of the membrane (*h *= *R*_2 _- *R*_1 _= 50 μm) and *L*_*p *_is the hydraulic permeability. This approximation of the radial momentum equation, also known as the *Starling's *law of filtration, is widely used in studies of membrane filtration [22]. It should be noted however, that equation (4) does not take into account diffusion or unsteady effects, while the *Brinkman *approximation, employed in the present study, does account for these contributions.

The mass transport of solute *i *is given by(5)

where *C*_*i *_and *D*_*i *_are the concentration and the diffusion coefficient of solute *i *in the mixture, respectively. In the free-flow domains, the retardation coefficient is *λ *= 1, while in the membrane region *λ *= 1 - *σ*, where σ is known as the reflection coefficient. It is a phenomenological parameter that characterizes the hindrance of convective transport across the membrane; for small molecule solutes, as considered in the present study, *σ *= 1 is assumed.

Referring to Figure 2, the following boundary conditions are employed. At the inlet the velocity and mass-fraction ratio are specified(6)

where *u *and *v *are the axial and radial velocity component, respectively.*C*_*i,B *_and *C*_*i,S *_are the concentration of solute *i *at the blood and dialysate inlets, respectively. Due to the low *Reynolds number *of the flow, a fully developed flow is assumed both at the blood and dialysate inlets. At the outlet, the pressure is satisfied:(7)

where *P*_*B*0_(*t*) and *P*_*S*0_(*t*) are the outlet pressure of the blood and dialysate domains, respectively. The value of the inlet velocity and outlet pressure waveforms are specified from the experimental measurements. Outflow conditions are specified for the mass-fraction ratio.

The domain of solution is meshed by Gambit (Ansys, Inc.) using quadrilateral cells with clustering near solid walls and near the membrane edges. A total of over 30,000 cells are employed based on a mesh-independence test. The solution of the finite-volume discrete equations is performed using Fluent (Ansys, Inc.). A second-order accurate scheme is used with the SIMPLE approach for decoupling the pressure.

### Physical Properties and Non-Dimensional Numbers

In the experimental investigations, imitation blood/isotonic saline is used as well as heparinized porcine blood. In the numerical simulations, a Newtonian aqueous solution (isotonic saline) is defined with density of *ρ *= 1000 kg/m^3 ^and constant viscosity of *μ *= 0.001 kg/m sec. In the dialysate, plasma is assumed to flow with the same properties. The mass diffusion coefficient in the blood and dialysate sides is assumed to be identical. The value of the mass transfer coefficient for each solute as well as the permeability are estimated from the experimental results as elaborated in the Results section.

Three non-dimensional numbers govern the flow and mass transfer processes in the model of the hollow fiber: the Reynolds (*Re*), Peclet (*Pe*) and the Womersley (*α*) numbers. These non-dimensional numbers are evaluated separately for the free flow domains and for the porous membrane domain. In the free flow domains, the Reynolds number (, where *d *is a reference length) is based on *d *= 2 *R*_1 _or *R*_3 _- *R*_2 _for the blood or dialysate domains, respectively, and *u *is a mean velocity. The Womersley number is given by , where *f *is the frequency of the inflow waveform; is the kinematic viscosity or the appropriate mass transfer coefficient. The *Peclet *number for solute *i *is calculated from: . In the porous membrane, the *Peclet *number can be estimated from: , where, *v *is the radial velocity component, *UF *is the ultrafiltration rate, *A *is the total surface (0.6 m^2^) of the hollow fibers and *D*_*i,m *_is the effective mass diffusivity of solute *i *in the membrane.

Table 1 presents separately for the free-flow and for the membrane domains the non-dimensional numbers for a flow rate of 100 ml/min and an ultrafiltration rate of 20 ml/min. These values correspond to the upper limits existing in the WAK. The mass diffusivity is assumed to be 10^-9 ^and 10^-10^m^2^/s in the free flow and membrane domains, respectively. The frequency of the WAK pump (delivering both the blood and the dialysate in counter-phased flows) is taken to be 2 Hz.

**Table 1:** The non-dimensional numbers (Mom. = momentum equation, mass = mass transfer equation)

			*α*	
	*Re*	*Pe*	*Mom*.	*Mass*
Free-flow	0.50	1200	0.43	22
Membrane	0	0.16	0.06	15

The Reynolds number is small in the fiber lumen (*Re *= 0.5) and negligible in the membrane, justifying the Brinkman approximation. The Peclet number is large in the free flow domains (*Pe *= 1200), indicating the dominance of mass convection in the blood and dialysate domains. However, in the membrane region, the mass transfer is dominantly viscous (*Pe *= 0.16). The Womersley number, that estimates the ratio of unsteady to diffusion terms, is relatively small (*α *= 0.4), indicating a quasi-steady flow. In the membrane region, the Womersley number is negligible, i.e., unsteady effects are negligible. The Womersley number related to the mass transfer processes is large, especially in the blood and membrane domains (*α *= 22 and 15, respectively). These large values correspond to a saturated flow, i.e. the mass transfer processes are actually steady (except in a very thin Stokes-like layer). Therefore, although the WAK pump induces time-dependent flow, the flow and mass transfer processes are quasi-steady and the performance of the dialyzer can be represented by the mean flow.

## Results

A large number of experimental cases have been considered differing in the pump types, blood and dialysate flow rates, ultrafiltration rate and the afterload. Table 2 summarizes the relevant experimental results. The flow and mass transfer processes are quasi-steady and therefore the mean of the inlet and outlet pressures and flow rates have been calculated from the time-dependent experimental results. The *TMP *is calculated using the approximation(8)

**Table 2:** Summary of the mean operating conditions of the experimental cases (P(mmHg), Q(ml/min), UF(ml/min), KUF(ml/hr/mmHg))

Case	***P***_***b,in***_	***P***_***b,out***_	***P***_***d,in***_	***P***_***d,out***_	***Q***_***b,in***_	***Q***_***b,out***_	***Q***_***d,in***_	***Q***_***d,ou***_	*TMP*	*UF*	***K***_***UF***_
Roller pump for blood, centrifugal pump for dialysate, with afterload											

1	108	100	166	172	83	100	45	28	-65	-17	16.0
2	71	63	158	162	49	76	54	26	-93	-28	18.0
3	58	50	112	116	46	65	38	18	-60	-20	20.0
4	40	33	88	89	28	49	33	14	-52	-20	23.0
5	136.	127	167	173	100	118	45	26	-39.	-18	28.0
6	162.	152	171	177	121	132	38	27	-17	-10	36.0
7	188	180	177	183	142	144	31	28	4	-3	-40.0

Roller pump for blood, centrifugal pump for dialysate, without afterload											

15	62	57	17	9	82	71	42	53	47	11	14.0
16	27	23	8	2	40	39	43	44	20	2	4.5
17	17	14	-1	-9	28	26	31	33	21	2	4.9
18	13	10	-4	-12	20	19	24	26	20	2	5.3
21	62	55	22	20	82	72	50	59	38	10	15.0
22	44	37	9	9	65	58	40	47	32	7	13.0
23	32	27	3	2	52	45	34	40	27	6	14.0
24	22	19	-6	-8	42	36	20	26	28	6	14.0
25	11	8	-13	-14	23	19	9	13	23	4	10.0

WAK pump for both blood and dialysate, with afterload											

31	39	33	-25	-32	77	51	42	65	64	24	23
32	24	21	-24	-31	50	34	38	56	50	17	20
33	19	16	-21	-29	37	25	33	44	42	12	16
34	6	5	-19	-25	10	4	30	35	27	5.5	12
35	22	18	-22	-30	43	29	36	50	46	14	18

WAK pump for both blood and dialysate, without afterload, porcine blood											

41	31	30	13	10	37	39	40	38	19	-2	-6
44	33	34	10	5	46	48	60	58	26	-2	-5
47	43	43	12	7	57	56	56	57	34	1	2
50	51	45	7	-1	81	81	112	112	45	0	0

Roller pump for blood, centrifugal pump for dialysate, without afterload, porcine blood											

53	34	32	24	20	44	46	51	49	11	-2	-13

56	36	35	26	24	51	54	67	64	11	-3	-15
59	39	36	27	25	60	62	79	77	12	-2	-12
62	55	48	34	27	82	84	112	110	21	-2	-7

where *P*_*b *_and *P*_*d *_are the mean blood and dialysate pressure and *in *and *out *refer to the inflow and outflow, respectively. The ultrafiltration coefficient *K*_*UF *_is calculated from:(9)

where *UF *is the ultrafiltration rate.

Cases 1-7 present the results obtained for the roller pump with the four canisters in place (i.e. with an afterload), while cases 15-25 refer to the roller pump cases, but with the canisters removed (i.e. without an afterload) and cases 31-35 employ the WAK pump with an afterload as well. These experiments are conducted using imitation blood, while in cases 41 through 62 porcine blood is used and no afterload is applied. It should be noted that in the roller pump experiments with the canisters in place, a significant backfiltration (negative ultrafiltration) is obtained. This is because the single roller pump *pumps *the dialysate *into *the dialyzer while the WAK pump *pulls *the dialysate *out of *the dialyzer, thus creating suction in the dialysate compartment. This suction effect increases the pressure difference between the blood and dialysate compartments and thus, enhances the convective mass transport.

The presentation of the results is divided into two parts, (i) ultrafiltration and (ii) clearance results.

### Ultrafiltration Characteristics

**Figure 3:**
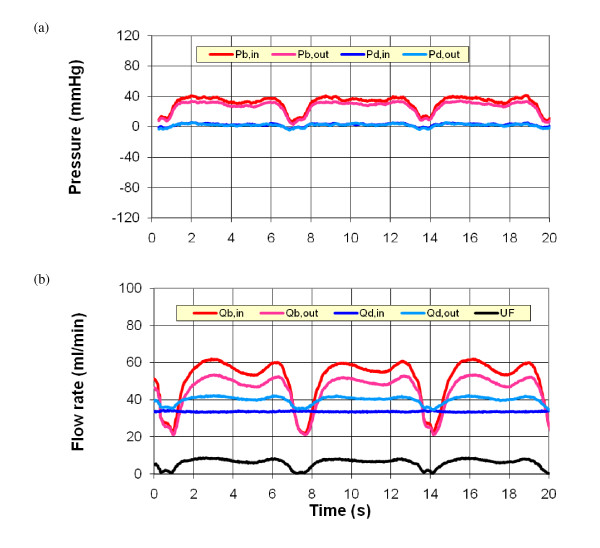
**Experimental measurements of the inlet and outlet (a) pressures and (b) flow rates for the roller pump with no afterload (case 23 in Table 2)**.

**Figure 4:**
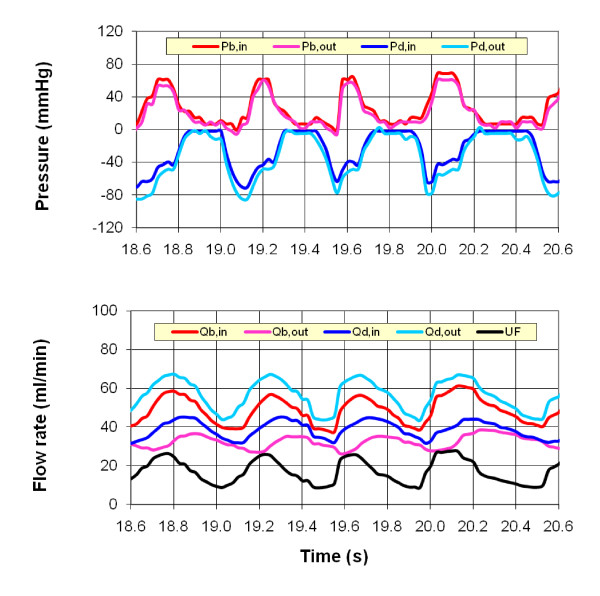
**Experimental measurements of the inlet and outlet (a) pressures and (b) flow rates for the WAK pump (case 32 in Table 2)**.

Figures 3 and 4 present sample experimental results for the two different pump setups used in the experiments, the roller pump and the WAK pump setups (cases 23 and 32 in Table 2), respectively. These cases are selected to have similar inflow rates. In each case, the pressures and flow rates at the inlet and outlet of the blood and dialysate compartments are shown, together with the ultrafiltration rate. The roller pump yields a quasi-steady flow, while the WAK pump generates an inherently time dependent flow. In the latter case, the pressure waveforms for the blood and dialysate compartments are in 180° phase due to the novel design of the WAK pump. Similar results have been obtained for other operating conditions of the two setups.

**Figure 5:**
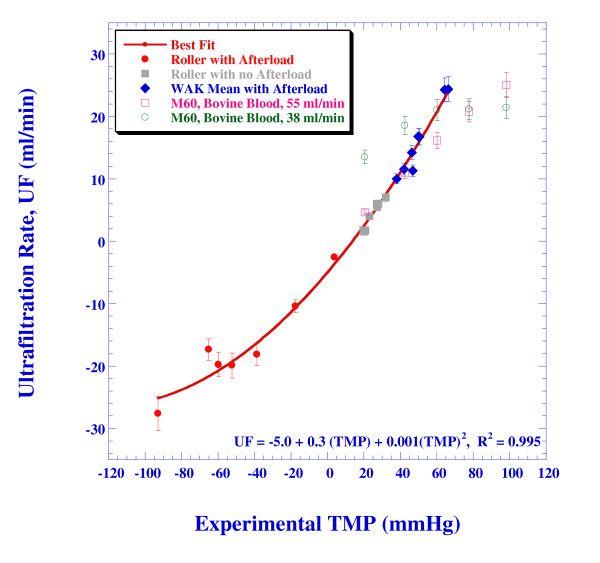
**The dependence of the ultrafiltration flow rate on the trans-membrane pressure (experimental results)**. The solid red line shows the best-fit to second order polynomial for the entire range of results. The manufacturer's results for the M60 dialyzer at 55 ml/min and 38 ml/min using bovine blood are also presented up to *TMP *= 100 mmHg.

Figure 5 depicts the dependence of the ultrafiltration rate on *TMP*. Each one of the above mentioned case-categories is marked by a different symbol: circles for the roller pump experiments with the afterload, squares for the roller pump experiments without afterload and the diamonds for the WAK pump experiments with afterload. Although the mean blood and dialysate flow rates vary from case to case (Table 2), Figure 5 reveals that the ultrafiltration rate depends mainly on the *TMP*. However, the dependence is nonlinear, contrary to present practices that assume a linear relationship, [22]. This is revealed by the best fit to a second-order polynomial of the roller-pump experimental data points and separately for the WAK pump experiments. The slopes obtained for the WAK pump are larger than that for the roller pump, but not by a significant margin. For the sakes of brevity, we refrain from displaying separate fits to the data points and instead we are presenting a good correlation (*R*^*2 *^= 0.995) to a second-order polynomial for the entire range of results, see the solid line in Figure 5.

Figure 5 also presents *UF *vs. *TMP *as published in the spec-sheet of Gambro's M60 [23] for two blood inflow rates relevant to the WAK operation (55 and 38 ml/min, see hollow squares and hollow circles, respectively). The agreement between the present experiments and Gambro's data is reasonable, but one has to note that the latter is obtained for bovine blood, while the present results refer to blood analogous liquid with vanishing osmotic pressure. The *UF *rate for aqueous solution should be larger by as much as 15% [14,16]. On the other hand, the actual *UF *data are worse than published by the manufacturers by about the same percentage [16], resulting in the good agreement.

**Figure 6:**
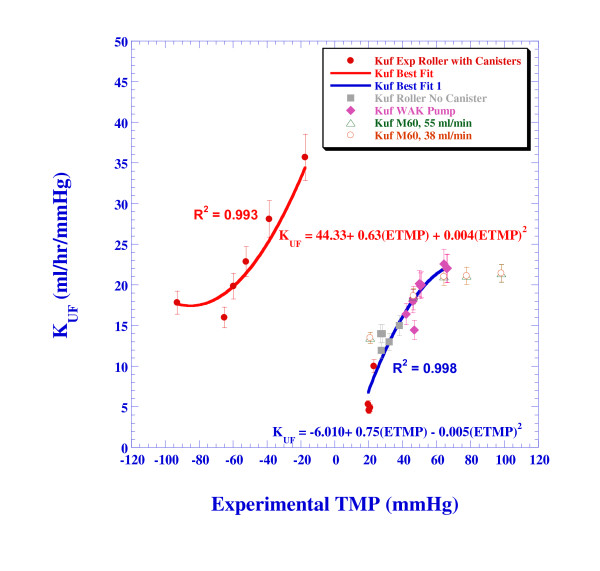
**The dependence of the ultrafiltration coefficient on the trans-membrane pressure (experimental results)**. Note that when roller pump is used with afterload, significant backfiltration (negative ultrafiltration) is obtained and thus, the *TMP *is negative whereas in other cases, the *TMP *is positive showing maximum values when the WAK pump is used.

The dependence of the ultrafiltration coefficient on the *TMP *is shown in Figure 6. For positive *TMP *(pressure in the blood is larger than in the dialysate), the ultrafiltration coefficient increases with *TMP*, although the increase rate slows down as the *TMP *increases. The asymmetry in the *K*_*UF *_for positive and negative *TMP *is very clear. Common practices assume that *K*_*UF *_is constant, independent of the *TMP*. This is indeed observed in the Gambro data of the M60 dialyzer (that is also shown in Figure 5) for the higher *TMP *range [23]. The M60 dialyzer of Gambro yields an ultrafiltration coefficient in the range of 16-22 ml/hr/mmHg for bovine blood, depending on the blood flow rate. However, for the lower range relevant to the present study (*TMP *< 40 mmHg), the Gambro data shows as well a dependence of the ultrafiltration rate on *TMP*. The Filtral 6 dialyzer-spec provides a *K*_*UF *_value of 13-17 ml/hr/mmHg for bovine blood, [23]. The present experiments results in a *K*_*UF *_in the range of 5 to 30 ml/hr/mmHg, depending on the *TMP *and its sign.

### Comparison with Numerical Simulations and Estimation of the Hydraulic Permeability

A set of numerical simulations have been performed, a simulation for every one of the cases of Table 2. The aim is to estimate the hydraulic permeability based on the experimental data. Common practices assume that the hydraulic permeability of a given dialyzer is constant, independent of the flow or pressure conditions. In the analysis of the experimental results, such an assumption has not been made and the hydraulic permeability, *L*_*p*_, (Eq. 4) is determined separately for each one of the experimental cases. For each one of the cases, *L*_*p *_(translated to porous resistance) was varied until the calculated *UF *rate was equal to the experimental value.

**Figure 7:**
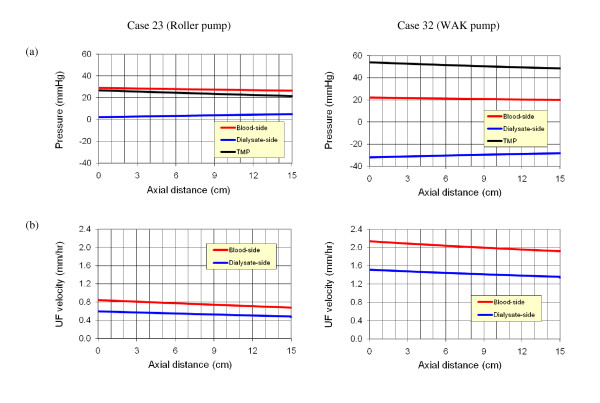
**The pressure and ultrafiltration velocity distribution along the blood and dialysate compartments of the dialyzer's membrane (computational results)**. The *TMP *induced by the WAK pump is about twice of the roller pump and so is the ultrafiltration velocity.

The calculated pressure variations and ultrafiltration velocity along the hollow fiber and along the dialysate are shown in Figure 7 for the two cases that have been already considered in Figures 3 and 4. The pressure distribution in the hollow fiber (blood side) is reasonably similar in both cases. However, in the dialysate compartment, large negative gauge pressure is created by the WAK pump whereas in the roller pump case, a small positive pressure is established. This difference generates a *TMP *twofold larger than in the roller pump case, with a similar increase in the ultrafiltration velocity. Consequently, the contribution of convection to the overall clearance of solutes increases substantially when the WAK pump is used.

**Figure 8:**
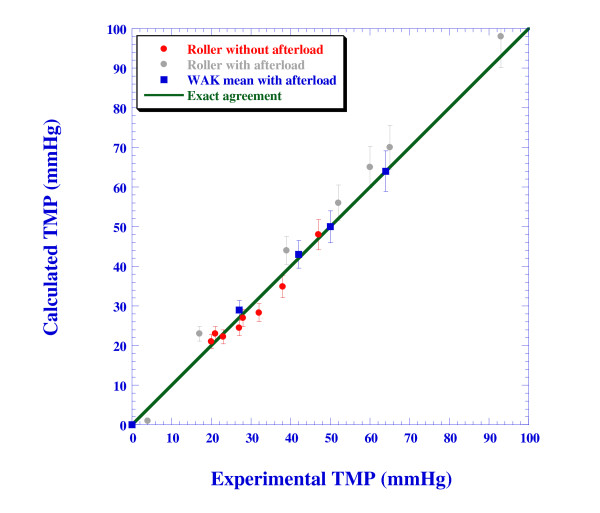
**Comparison of the calculated and the experimental *TMP***.

Based on the calculated inlet pressure and the imposed outlet pressure, the *TMP *is calculated for all the numerical cases as well and the results are compared in Figure 8 with the experimental values. The line marking a perfect match is also shown. The numerical results over-predicted the roller pump *TMP *by 3-6 mmHg. The agreement with the WAK pump experiments is excellent with a deviation of less than 1.5 mmHg. These small deviations can be attributed to experimental errors that are estimated to be ±5-10 mmHg as well as to pressure losses at the entrance and exit of the dialyzer. Pressure losses due to the non-uniform arrangement of the fibers in the dialyzer have not been accounted for in the present numerical model.

The ultrafiltration rate can be calculated from the *TMP *using the Starling's law without osmotic pressure effects (since no proteins are present): *UF *=*L*_*p*_*A*·*TMP*, where *A *is the total fiber, yielding,(10)

**Figure 9:**
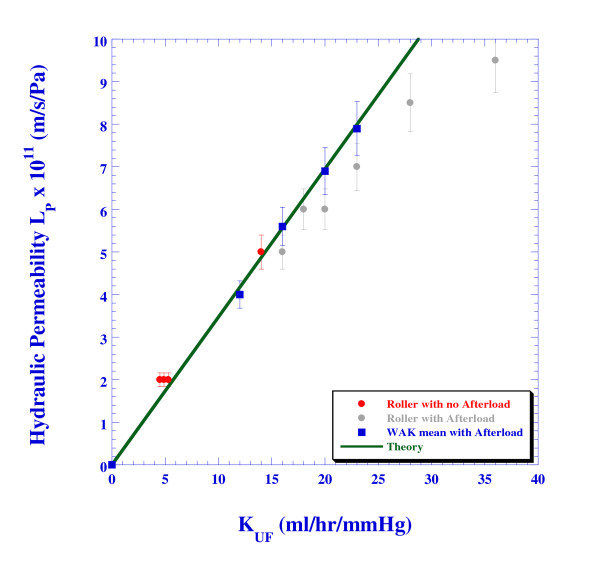
**The dependence of the hydraulic permeability on the ultrafiltration coefficient KUF**.

Figure 9 depicts the relationship between the calculated *L*_*p *_and the *K*_*UF *_as found from equation (9) using the experimental values of the ultrafiltration rate and *TMP*, together with the line that plots the relationship (10) (marked as "Theory"). A significant deviation from the theoretical value is obtained only for ultrafiltration coefficients higher than 25 ml/hr/mmHg for the roller pump case with an afterload, when significant backfiltration is obtained. However, for *K*_*UF *_relevant to the WAK operation, the Starling's law describes accurately the linear relationship between the ultrafiltration rate and the hydraulic permeability.

### Clearance Results

The concentrations of urea, creatinine and other solutes are measured experimentally at the inlet and exit of both the blood and dialysate compartments. Table 3 summarizes the measured concentration of BUN (representing urea) and creatinine for selected roller pump and the WAK pump setups.

**Table 3:** Summary of the urea and creatinine concentrations at the inlet and exit as obtained from experiments. Roller pump without afterload is used in cases 21-25 and the WAK pump (with afterload) is used in cases 31, 32 and 35

	BUN (mg/dL)		Creatinine (mg/dL)	
	
	Blood	Dialysate	Blood	Dialysate
Inlet (mg/dL)	118	0	4.5	0

Case 21	73	95	3.0	3.1
Case 22	68	95	2.7	3.1
Case 23	56	88	2.3	2.9
Case 24	60	100	2.4	3.5
Case 25	61	99	2.4	3.6

				

Inlet (mg/dL)	38	0	6.8	0

Case 31	24	31	4.9	5.1
Case 32	8	21	2.1	3.5
Case 35	4	18	0.7	3.2

The mass diffusion coefficient of each solute in the blood, dialysate and membrane regions is determined based on the results of case 23. In the cases elaborated in Table 3, imitation blood is used and therefore it is reasonable to assume that the mass diffusion coefficients of the aqueous blood and the dialysate are identical. The blood and dialysate mass transfer coefficients of the free-flow and membrane flow are varied (separately for urea and creatinine), within the range of values found in the literature, until the calculated exit concentration of both the blood and dialysate matches the experimental results. An excellent match has been obtained for a mass transfer coefficient of *D *= 1.5·10^-9 ^*m*^2^/*s *and 1.3·10^-9 ^m^2^/s for urea and creatinine in the free-flow regions, and *D *= 1.3·10^-10 ^*m*^2^/*s *and 1·10^-10 ^*m*^2^/*s *in the membrane regions, respectively. These values of the mass transfer coefficients are used in the numerical simulations of the other cases. In vitro results by Sakai [24] using water found mass transfer coefficient of *D *= 1.8·10^-9 ^*m*^2^/*s *and 1.3·10^-9 ^m^2^/s for urea and creatinine, respectively. Cussler [25] cites the value of 1.38·10^-9 ^m^2^/s for urea in aqueous solution. The corresponding mass transfer coefficients for blood are found in vivo to be *D *= 1.4·10^-9 ^m^2^/s and 0.83·10^-9 ^m^2^/s, respectively.

**Figure 10:**
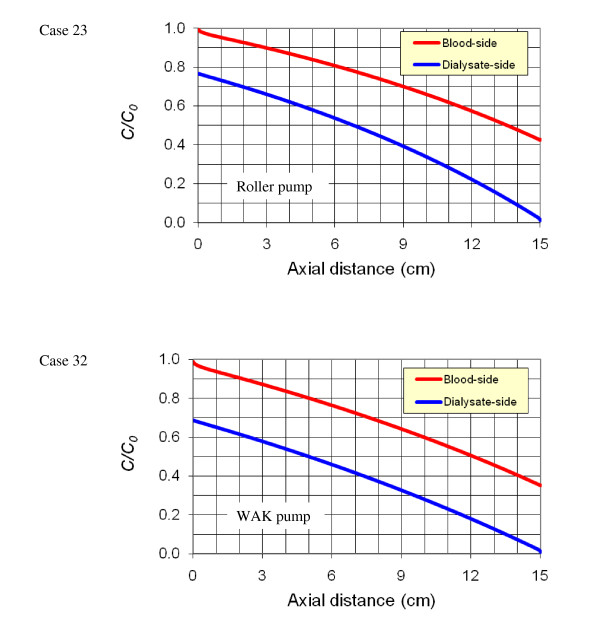
**Comparison of the distribution of the relative urea concentration along the two edges of the membrane for the roller pump versus the WAK pump (computational results**. The use of the WAK pump resulted in approximately 20% more urea removal than the roller pump.

**Figure 11:**
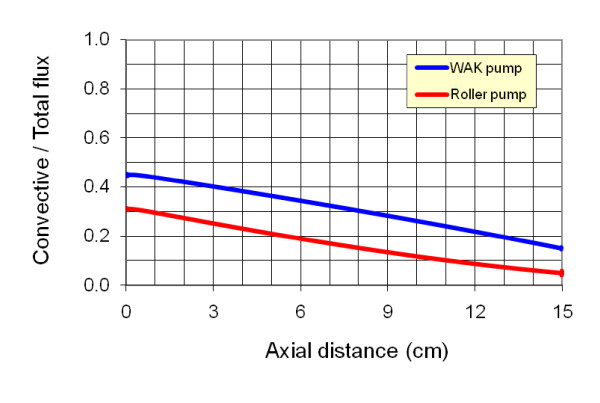
**The distribution of the ratio of the convective urea transfer to the total urea transfer along the membrane for the two sample cases 23 (roller pump) and 35 (WAK pump)**.

The distributions of the calculated urea concentration, *C*, relative to the inlet concentration (*C*_*0*_) along the two sides of the membrane are shown in Figure 10 for cases 23 (roller pump) and 32 (WAK pump). The distributions of the pressure and ultrafiltration velocity for these cases have been presented in Figure 7. Obviously, the setup with the WAK pump (case 32) shows a better clearance of the urea. The reason is attributed to the higher ultrafiltration rate obtained with the WAK pump. This higher ultrafiltration rate increases the convective mass transfer flux, as presented in Figure 11. In the entrance to the hollow fiber, the convective mass ratio is as high as 45% and 30% of the total mass transfer for the WAK and roller pump cases, respectively, while at the exit, the ratio decreases to 15% and 5%, respectively. In the WAK pump case, the overall convective urea transfer ratio is 31% of the total urea transfer, while in the roller pump case it is 17% only.

The ultrafiltration rate in the WAK cases is large relative to the blood flow rate (30-50%) and therefore the conventional definition of the clearance is inappropriate. In these cases, we suggest to define clearance by(11)

where *Q*_*B *_is the blood volumetric flow rate *C*_*B *_and is the concentration of the solute in the blood. In the case of low ultrafiltration rate *Q*_*B,in *_≈ *Q*_*B,out *_and the definition (11) is identical to the conventional definition of clearance. The clearance results for both the experiments and the numerical simulations are summarized in Table 4. The agreement between the experimental and the numerical results is good for the roller pump and reasonable for the WAK pump. The calculated errors between the experimental and numerical results are within the expected range given that the experimental procedures suffer from various measuring error sources of the flow rate, pressure and concentrations. These estimated error values are 7-10%, 10-15% and 5-7%, respectively. Please note that in the low flow rate cases, the flow and pressure measurements are noisier than in the higher flow rates and thus, the errors are higher for the low flow.

**Table 4:** Comparison of the experimental and calculated clearance data for urea and creatinine

	Urea Clearance (ml/min)			Creatinine Clearance (ml/min)		
**Case #**	**Exp.**	**Num.**	**Err (%)**	**Exp.**	**Num.**	**Err (%)**

21	37	40	-8	24	26	-8
22	31	34	-9	21	22	-5
23	30	30	0*	20	20	0*
24	24	24	0	16	16	0
25	13	12	8	9	8	11

31	44	48	-8	28	32	-13
32	43	37	14	28	25	11
35	40	34	15	28	22	21

41	25	29	-14	16	18	-11
44	32	35	-9	21	22	-5
47	39	40	-3	24	25	-4
50	52	51	2	31	30	3

53	28	32	-13	19	21	-10
56	35	33	6	22	20	9
59	40	41	-2	25	24	4
62	52	50	4	31	30	3

*The mass transfer coefficients have been determined from this case

**Figure 12:**
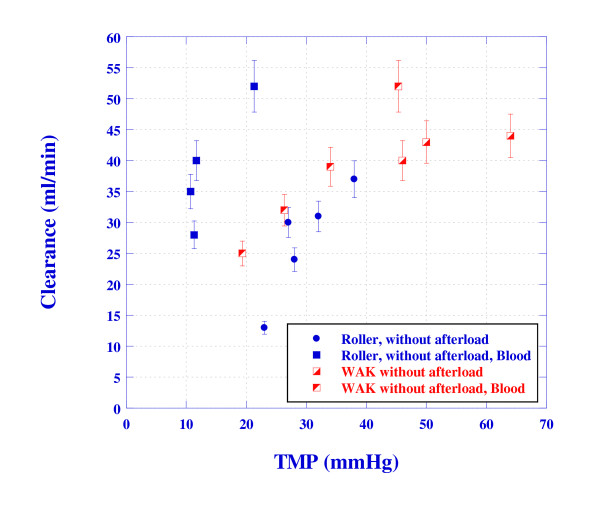
**The dependence of the urea clearance on TMP for the WAK and roller pump setups, irrespective of other parameters**.

The experimental urea clearances versus the *TMP *using the WAK and roller/centrifugal pumps are plotted in Figure 12 irrespective of the other parameters (such as flow rates). The creatinine clearance shows a very similar trend. Obviously, the WAK setup yields similar or better clearance than the roller pump setup. The apparently only exception is the roller pump case using porcine blood without afterload (cases 53-62). However, a closer look reveals that the better clearance is obtained because of the increased dialysate flow rate by as much as a factor of two in these cases, see Table 2.

## Discussion

The experimental and numerical investigations of this study have shown the impact of pulsatile flow in transport of solutes in a high-flux dialyzer. The parameters influencing the transport phenomena across the dialyzer membrane in counter-phased pulsatile flow in the blood and dialysate compartments are studied. The findings support the historical belief of dialytic-therapy pioneers who designed dialyzers based on counter-current exchangers [1,2] to exploit diffusive mass transport phenomena of small molecules. However, the present study also reveals the relative contribution of ultrafiltration in to enhanced clearance performance. These findings corroborate with other researchers' findings on the advantages of 'solvent drag' and push-pull mechanisms [3-8]. These latter studies point to the effect of pulsatile blood flow on the filtration rate of hollow fibers [4,7,8] and adsorption of proteins on these hollow fibers [9-13]. The indicated effective blood purification is believed to result from convective solute removal and protein washout by the push-pull mechanism. A push-pull hemodiafiltration (HDF) device provides rapid forward-backward filtration alternately [8,9], leading to alternate flow of body fluid and sterile pyrogen-free dialysate across a high flux hollow-fiber membrane. These conventional push-pull HDF devices, however, need a disposable blood-volume-control bag and can have dangerously high trans-membrane pressures (TMP). These potentially unsafe conditions can be avoided by using ultrafiltrate volume controllers and rigid synthetic hollow fibers such as polyacrylonitrile, polysulfone, and polyamide [8,9].

The present study has shown experimentally that these hazardous conditions do not exist in the WAK system. The *TMP*'s are always within safe ranges of hollow-fiber mechanical strength and there is no need for blood volume control. Furthermore, our studies have produced reliable information on flow/pressure behavior of blood and dialysate passing through relevant dialyzer compartments, as well as information on clearance of urea and creatinine, for both the WAK pump and the roller/centrifugal pump pair, delivering blood and dialysate at comparable flow.

The impact of afterload generated by a set of powder-filled canisters on the ultrafiltration was thoroughly studied and compared with the same system without the afterload (no canisters). The results have shown that when the WAK pump was used, the counter-phased blood and dialysate flows created twice as much pressure difference across the membrane than that of the roller-centrifugal pumps (Figure 9). The results summarized in Table 1 and depicted in Figure 7 have indicated that the trans-membrane pressure is increased with the ultrafiltration rate. However, this relationship was not found to be linear but rather corresponds with a second-order polynomial function. The numerical model re-established the linear relationship between the hydraulic permeability and the ultrafiltration coefficient.

One of the major findings of the present study has been attributed to the efficiency of the WAK pump in removing urea from blood. Figure 12 depicted better clearance of urea when the WAK pump was used as oppose to conventional roller pump designs. The reason for this phenomenon is attributed to the higher ultrafiltration rate obtained with the WAK pump. This higher ultrafiltration rate caused by the higher *TMP *increases the convective mass transfer flux along the length of the fiber. With the WAK pump, the convective urea transfer is 31% of the total urea transfer, while with the roller pump that value is only 17%.

## Conclusions

It has been shown that the small, light-weight, battery-operated WAK pump yields the same or slightly better clearances of urea and creatinine as the large, heavy, AC power-requiring roller pump. Therefore, the dual-channel pulsatile pump is the most suitable configuration for use in conjunction with the wearable artificial kidney while saving energy and delivering adequate blood and dialysate flows for around-the-clock dialysis.

To further expand these findings, additional studies are required to determine the efficiency of the WAK pump in removing other solutes such as phosphor, potassium, calcium, beta-2-microglobulin or Para-cresol. Moreover, some of the computational model assumptions, such as axisymmetry and uniform inlet flow conditions into the fibers should be relaxed.

## Competing interests

ER & MB were supported by BioQuantetics Inc. under contractual agreement with National Quality Care Inc (NQCI) of Beverly Hills, CA. There has been no financial support from NQCI. MR was awarded by NQCI PC time for the execution of the calculations. ER & MB hold 10K NQCI stocks. There has been a patent application filed by ER based on the content of the manuscript. The patent filing fees have been paid by the organization (NQCI). There are no any other financial or competing non-financial interests.

## Authors' contributions

ER and MB have designed the experimental setup, conducted the experiments and their analyses. MR performed the numerical simulations and analyses. All authors were actively involved in the writing of the manuscript, read it and approved the final manuscript.

## References

[B1] Alwall N On the artificial kidney. I. Apparatus for dialysis of blood 'in vivo' Acta Med Scand 1947 128 317 321 18897403

[B2] Kolff WJ First clinical experience with the artificial kidney Ann Intern Med 1965 62 608 612 10.7326/0003-4819-62-3-608 14263109

[B3] Henderson LW Besarab A Michaels A Bluemle LW Blood purification by ultrafiltration and fluid replacement (diafiltration) Trans Am Soc Artif Intern Org 1967 17 216 221 10.1111/j.1492-7535.2004.00081.x 19379396

[B4] Ding L Laurent JM Jaffrin MY Dynamic filtration of blood: A new concept for enhancing plasma filtration Int J Artif Organs 1991 14 365 370 1885245

[B5] Pedrini LA De Cristofaro V On-line mixed hemodiafiltration with a feedback for ultrafiltration control: Effects on middle-molecule removal Kidney Int 2003 64 1505 1513 10.1046/j.1523-1755.2003.00240.x 12969172

[B6] Ronco C Ghezzi PM Brendolan A Crepaldi C La Greca G The hemodialysis system: Basic mechanisms of water and solute transport in extracorporeal renal replacement therapies Nephrol Dial Transplant 1998 13 Suppl 6 3 9 10.1093/ndt/13.suppl_6.3 9719196

[B7] Jaffrin MY Ding LH Gupta BB Rationale of filtration enhancement in membrane plasmapheresis by pulsatile blood flow Life Support Systems 1987 5 267 271 3695585

[B8] Shinzato T Fujisawa K Nakai S Miwa M Kobayakawa H Takai I Morita H Maeda K Newly developed economical and efficient push/pull hemodiafiltration Contrib Nephrol 1994 108 79 86 8039400 10.1159/000423360

[B9] Maeda K Shinzato T Push/pull hemodiafiltration Contrib Nephrol 2007 158 169 176 10.1159/000107247 17684355

[B10] Morti SM Zydney AL Protein-membrane interactions during hemodialysis: Effects on solute transport ASAIO J 1998 44 319 326 10.1097/00002480-199807000-00016 9682960

[B11] Sun S Yue Y Huang X Meng D Protein adsorption on blood-contact membranes J Membr Sci 2003 222 3 18 10.1016/S0376-7388(03)00313-2

[B12] Valette P Thomas M Dejardin P Adsorption of low molecular weight proteins to hemodialysis membranes: Experimental results and simulations Biomaterials 1999 20 1621 1634 10.1016/S0142-9612(99)00070-8 10482417

[B13] Moussy Y Bioartificial Kidney. I: Theoretical analysis of convective flow in hollow fiber modules: Application to a bioartificial hemofilter Biotech Bioeng 1999 68 142 152 10.1002/(SICI)1097-0290(20000420)68:2<142::AID-BIT3>3.0.CO;2-R 10712730

[B14] Eloot S Watcher DD Tricht IV Verdnock P Computational flow modeling in hollow-fiber dialyzers Artificial Organs 2002 26 590 599 10.1046/j.1525-1594.2002.07081.x 12081517

[B15] Liao Z Poh CK Hardy PA Clark WR Gao D A numerical and experimental study of mass transfer in the artificial kidney J Biomech Eng 2003 125 472 480 10.1115/1.1589776 12968571

[B16] Eloot S Vierendeels J Verdonck P Optimization of solute transport in dialyzers using a three-dimensional finite volume model Comput methods Biomed Engin 2006 9 363 370 10.1080/10255840601002728 17145670

[B17] Gura V Beizai M Ezon C Polaschegg HD Ronco C, Brendolan A, Levin NW Continuous renal replacement therapy for end-stage renal disease: The wearable artificial kidney (WAK) Contrib Nephrol 2005 149 Cardiovascular Disorders in Hemodialysis, Basel, Karger 325 333 full_text 15876856 10.1159/000085694

[B18] Davenport A Gura V Ronco C Beizai M Ezon C Rambod E A pilot study of a wearable artificial kidney in end stage renal failure patients Lancet 2007 370 2005 2010 10.1016/S0140-6736(07)61864-9 18083402

[B19] Gura V Ronco C Davenport A The wearable artificial kidney, why and how: from Holy Grail to reality Seminars in Dialysis 2009 22 13 17 10.1111/j.1525-139X.2008.00507.x 19000114

[B20] Gura V Macy AS Beizai M Ezon C Golper TA Technical breakthroughs in the wearable artificial kidney (WAK) Clin J Am Soc Nephrol 2009 4 1441 1448 10.2215/CJN.02790409 19696219 PMC2736696

[B21] Gura V Davenport A Beizai M Ezon C Ronco C β2-Microglobulin and phosphate clearances using a wearable artificial kidney, a pilot study Am J Kidney Dis 2009 54 104 111 10.1053/j.ajkd.2009.02.006 19376616

[B22] Bird RB Stewart WE Lightfoot EN Transport Phenomena 2002 2 John Wiley & Sons, Inc

[B23] Gambra - Filtral-AN69 spec 2008 http://www.gambramedical.ru/wmc/en/en_dialysis/en_dialysisproduct/en_dialyzers/en_filtral/

[B24] Sakai K Determination of pore size distribution 2. Dialysis Membranes J Membrane Sciences 1994 96 91 130 10.1016/0376-7388(94)00127-8

[B25] Cussler EI Diffusion: Mass Transfer in Fluid Systems 1997 2 Cambridge University Press

